# PVA-Microbubbles as a Radioembolization Platform: Formulation and the In Vitro Proof of Concept

**DOI:** 10.3390/pharmaceutics15010217

**Published:** 2023-01-08

**Authors:** Valerio Da Ros, Letizia Oddo, Yosra Toumia, Eugenia Guida, Silvia Minosse, Lidia Strigari, Silvia Strolin, Giulia Paolani, Francesca Di Giuliano, Roberto Floris, Francesco Garaci, Susanna Dolci, Gaio Paradossi, Fabio Domenici

**Affiliations:** 1 Department of Biomedicine and Prevention, University Hospital of Rome “Tor Vergata”, 00133 Rome, Italy; 2 Department of Chemical Science and Technologies, University of Rome “Tor Vergata”, 00133 Rome, Italy; 3 UOC Diagnostica per Immagini, University Hospital of Rome “Tor Vergata”, 00133 Rome, Italy; 4 Department of Medical Physics, IRCCS Azienda Ospedaliero—Universitaria di Bologna, 40138 Bologna, Italy

**Keywords:** PVA microbubbles, glioblastoma, radioembolization, yttrium, DOTA, RGD, HUVEC

## Abstract

This proof-of-concept study lays the foundations for the development of a delivery strategy for radioactive lanthanides, such as Yttrium-90, against recurrent glioblastoma. Our appealing hypothesis is that by taking advantage of the combination of biocompatible polyvinyl alcohol (PVA) microbubbles (MBs) and endovascular radiopharmaceutical infusion, a minimally invasive selective radioembolization can be achieved, which can lead to personalized treatments limiting off-target toxicities for the normal brain. The results show the successful formulation strategy that turns the ultrasound contrast PVA-shelled microbubbles into a microdevice, exhibiting good loading efficiency of Yttrium cargo by complexation with a bifunctional chelator. The selective targeting of Yttrium-loaded MBs on the glioblastoma-associated tumor endothelial cells can be unlocked by the biorecognition between the overexpressed α_V_β_3_ integrin and the ligand Cyclo(Arg-Gly-Asp-D-Phe-Lys) at the PVA microbubble surface. Hence, we show the suitability of PVA MBs as selective Y-microdevices for in situ injection via the smallest (i.e., 1.2F) neurointerventional microcatheter available on the market and the accumulation of PVA MBs on the HUVEC cell line model of integrin overexpression, thereby providing ~6 × 10^−15^ moles of Y90 per HUVEC cell. We further discuss the potential impact of using such versatile PVA MBs as a new therapeutic chance for treating glioblastoma multiforme recurrence.

## 1. Introduction

Glioblastoma multiforme (GBM) is the most common primary malignancy of the central nervous system, and it still represents an unsolved clinical problem with a median life expectancy of 15 months and an overall survival rate of 6.8% for patients at 5 years [1]. Surgical resection, followed by chemo-radiotherapy and adjuvant chemotherapy, represents the standard of care [2], but in patients with recurrent GBM, reoperation and second-line chemotherapy are critical and mostly suboptimal [3]. Furthermore, in the case of re-irradiation of the tumor mass, higher doses are needed to treat radioresistant GBM [4]. Stereotaxic brachytherapy and radiosurgery are more effective but are burdened by greater neurotoxicity. Moreover, transarterial radioembolization with Yttrium-90 (Y90-RE) has been used for treating primary and metastatic human liver cancer for over 20 years and has been proven effective [5]. The translation of this minimally invasive and super-selective radiotherapy approach on the brain has recently been performed as a proof-of-concept on canine models, demonstrating Y90-RE’s technical feasibility and safety, with initial encouraging data on its efficacy as a potential treatment for brain cancer [6]. However, data on this approach remain unique.

From then on, the research on novel groundbreaking strategies of personalized Y90-RE treatments with radioisotopes has dealt at first with the challenging formulation of suitable Y90 delivery carriers [7]. Specifically, an increasing effort from chemists has been dedicated to the realization of theranostic platforms exhibiting: (i) a good loading capacity of Y90; (ii) an in situ Y90 delivery compatible with the modern locoregional perfusion methods using a microcatheter [8]; (iii) the selective accumulation in the tumor vascular tissue, even including the possibility of modulating the release of Y90 targeted at the tumor interface by external stimuli [9]; and (iv) a multimodal detection of the carrier containing radioactive Y densified in the areas of the tumor vascularization or biodistributed in the human body [10].

In this framework, the development of echogenic microbubbles (MBs), with biocompatible polymeric shells, has redefined the ultrasound contrast agents [11] as intriguing drug delivery carriers of theranostic impact. Due to their micrometric dimensions, MBs remain within the vascular system. For active targeting, MBs are designed to selectively adhere to cell epitopes and receptors overexpressed on the endothelium associated with pathologies [11,12]. Compelling evidence in vitro and in vivo for their usage in designing a safe and non-invasive approach for selective and ultrasound-assisted drug delivery has been shown [12]. The MBs’ stimuli by ultrasound can indeed create echographic input information, together with a reversible opening on cell membranes and vessel walls (i.e., sonoporation), allowing a better transport of therapeutic agents through these natural barriers [11]. In particular, during the last decade, crosslinked-poly (vinyl alcohol) (PVA) MBs that functionalized with ligands are undergoing several investigations as an invaluable contrast agent and drug delivery carrier [13,14]. The major advantages of the PVA MBs as ultrasound-active theranostic agents, supporting both diagnosis and therapy, can be found in the robustness of the elastomeric shell, together with their chemical versatility in modifying the MBs with ligands and/or drugs at the water interface of the MBs’ surface [13,15,16].

Bearing these aspects in mind, we present herein the formulation of PVA-based MBs that allow us to obtain a microcarrier of radioactive Y90, which would be useful when directed, super selectively via the catheter, against the GBM vascular tissue. For this purpose, we engineered the crosslinked PVA shell of the MBs with the bifunctional chelator S-2-(4-Isothiocyanatobenzyl)-1,4,7,10-tetraazacyclododecane tetraacetic acid (p-SCN-Bn-DOTA, Macrocyclics, Plano, TX, USA) to load Y. The loading of Y by complexation with the chelator DOTA on the surface of MBs was successfully achieved and quantified using the non-radioactive isotope Y89 (which guarantees the same binding yield as Y90, while avoiding the risk of unnecessary exposure to ionizing radiation). We performed in vitro experiments to test the compatibility of the PVA MBs with one of the smallest 1.2F outer diameter neurointerventional flow-directed microcatheters available on the market (Magic, Balt).

It is known that invasive growth and angiogenesis in malignant gliomas involve the reprogramming of endothelial cells, including the increased expression of integrins α_v_β_3_ [17]. These heterodimeric trans-membrane proteins bind several extracellular matrix (ECM) ligands featuring an exposed NH2-Arginine-Glycine-Aspartic acid (‘‘RGD’’) sequence [18]. These integrins are highly distributed not only on the tumor-invasive endothelia but also on gliomas, in which they play a crucial role in cell–cell adhesion and cell–ECM interactions [19,20,21,22]. In this context, integrins α_v_β_3_ represent an important target for imaging gliomas and perturbing tumor-induced angiogenesis. 

In this work, we also demonstrate that the PVA MBs, as a Y90 carrier, can be derivatized with a Cyclo(Arg-Gly-Asp-D-Phe-Lys) (cRGD-Lys) ligand peptide, enabling the specific interaction with the integrin receptors overexpressed on the endothelium cell model HUVEC. Specifically, HUVEC is an endothelial cell line isolated from the umbilical cord vein. This cell line can be used in a variety of applications concerning cardiovascular diseases, including angiogenesis. In particular, HUVEC cells are currently used for the development of a three-dimensional vascularized in vitro tumor model in order to study tumor angiogenesis and cancer invasion [23,24]. Accordingly, HUVEC cells were screened for integrins α_v_β_3_ expression by immunofluorescence, and then used as a model for targeting glioma-associated vasculature via cRGD [18,19,23,24]. We successfully demonstrated that cRGD linked to the PVA MBs can be injected via catheter within a microchannel coated with HUVEC cells and can target the integrins of HUVEC in a vein-like fluid condition. Therefore, the PVA MBs can be used to locate Y via the catheter onto the endothelium surface by DOTA and cRGD, both coloaded onto the PVA MBs. This means that a super-selective microcatheter localization of PVA-DOTA MBs in the vascular tumor tissue could be further enhanced with a formulation of the carrier co-functionalized with cRGD.

## 2. Materials and Methods

### 2.1. Poly(Vinyl Alcohol) Microbubbles Preparation

PVA MBs were synthesized, following the procedure described elsewhere [15]. Briefly, fully hydrolyzed PVA (Sigma-Aldrich, Milano, Italy) 2% *w*/*v* aqueous solution in MilliQ water (18.2 MΩ.cm, PureLAB, Milano, Italy) was selectively oxidized using sodium metaperiodate (NaIO_4_, Sigma-Aldrich, Milano, Italy) at the vicinal hydroxyls groups to introduce aldehydes moieties. The subsequent crosslinking reaction between hydroxyls and aldehydes groups by acetalization was carried out at the water/air interface under vigorous stirring using an Ultra-Turrax homogenizer (IKA-instruments T25, Wertheim, Germany), generating an emulsion of MBs made out of an air core and a crosslinked PVA shell. The obtained MBs were washed from unreacted polymers and solid debris by flotation in separatory funnels, followed by centrifugation, for replacing the subnatant with water, and were then stored at room temperature (RT) for further functionalization. The size of the produced PVA MBs was 4.3 ± 0.7 μm (mean value ± standard deviation accounting for a population of 200 MBs), as determined by measuring the outer average diameter of fluorescein isothiocyanate (FITC isomer I, Sigma-Aldrich, Milano, Italy) tagged MBs by confocal laser scanning microscopy [16] (CLSM, Nikon Inverted Microscope Eclipse Ti-E, equipped with a Spectra-Physics Ar ion laser as light source at 488 nm, a Plan Apo 60× oil immersion objective with NA = 1.4, Nikon, and the Nikon software EZ-C1 3.9, image pixels 1024 × 1024 corresponding to a pixel size of ~200 nm). Further, the concentration of PVA MBs was determined in terms of number density (MBs/mL) by optical microscopy with a 40× long distance objective (Nikon Inverted Microscope Eclipse Ti-E, Newton, NJ, USA) using a cell counting chamber (Neubauer, Darmstadt, Germany). The optical images of MBs were recorded using Nikon EZ-C1 (version 3.9) software and analyzed by ImageJ freeware for the automated counting of the MBs within the smaller squares (0.25 × 0.25 mm^2^, 0.1 mm thickness) of the chamber, averaging from four different images. Then, the number density of the MBs was achieved according to Equation (1):

(1) $$\frac{\mathrm{Number}\;\mathrm{of}\;\mathrm{counted}\;\mathrm{MBs}\;\times 1000}{0.00625\;{\mathrm{mm}}^{3}\;\times \;\mathrm{dilution}\;\mathrm{factor}}$$

Moreover, Rhodamine B isothiocyanate (RBITC, Sigma-Aldrich, Milano, Italy) was used for the fluorescent tagging of the MBs to allow the imaging by fluorescence microscopy of MBs perfusing the channel from the microcatheter. For the labeling reaction, 2 μL of 0.5 mM dye solution in dimethyl sulfoxide (DMSO, Sigma-Aldrich, Milano, Italy) was added to an MB suspension (5 × 10^8^ MBs/mL), and the reaction was carried out for 1 h at RT in the dark. Then, RBITC MBs were washed by centrifugation (1000 rpm, 5 min) and resuspended in Milli-Q water several times, until the excess dye was removed.

### 2.2. PVA MBs Functionalization with cRGD Peptide

**Scheme 1:**
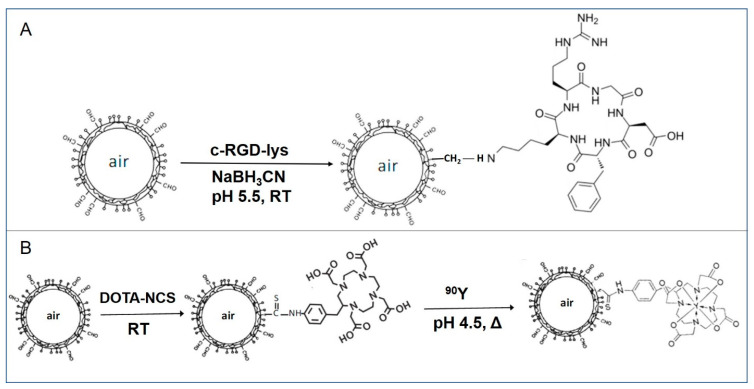
PVA MBs functionalization with cRGD peptide (**A**) PVA MBs functionalization with DOTA and yttrium (**B**).

In order to obtain cRGD-PVA MBs, cRGD peptide with a cyclic sequence of (Arg-Gly-Asp-D-Phe-Lys) (MedChemExpress, Monmouth Junction, NJ, USA) was used, coupled by reductive amination, according to Scheme 1, panel A. The PVA MB aqueous suspension was mixed with an excess amount of cRGD corresponding to a ratio of 0.2 μmol/10^8^ MBs (final reaction volume 2 mL). The reaction was carried out in acetate buffer (0.03 M CH_3_COOH and 0.02 M CH_3_COONa, pH = 5.0, Sigma-Aldrich, Milano, Italy) followed by the addition of 7 mg NaBH_3_CN (Merck). The MB suspension was slowly stirred at RT overnight using a vortex at 500 rpm. Then, the resulting MBs conjugated with cRGD were finally washed with MilliQ water using several steps of centrifugation. The amount of cRGD tethered on the MBs’ shell was estimated by spectrofluorimetric analysis of the unreacted peptide in the subnatant. The measurement was performed using a multimode plate reader (Spark^®^, Tecan, Männedorf, Switzerland) equipped with a UV transparent 96 well plate (Corning, Corning, NY, USA), and it was compared with the results from the peptide solutions with a known concentration (linear range 0.25–0.025 mg/mL). Each well of the plate was filled with 200 µL of the sample; the excitation wavelength was set to 235 nm, the emission to 283 nm, the gain to 100, the number of flashes to 30, and the integration time to 40 µs. Further, the z-position was optimized using the well containing the highest peptide concentration. The amount of peptide/MB was then assessed by determining the number density of MBs in the sample by optical microscopy.

### 2.3. PVA MBs Functionalization with DOTA and Y90 Loading

The preparation of the Y90 carrier consists of: (i) the functionalization of PVA MBs with DOTA (PVA-DOTA MBs); and (ii) Y90 loading. At this point, it should be stressed that all the preliminary experiments on Yttrium loading and labeling efficiency were carried out using the stable isotope Y89 in the form of Yttrium chloride (YCl_3,_ Sigma-Aldrich, Milano, Italy), thus avoiding the risk of exposure to ionizing radiation. A total amount of 10^9^ PVA MBs dispersed in MilliQ water reacted with 2 mg (2.90 μmol) of the bifunctional chelator p-SCN-Bn-DOTA, which was previously dissolved in 100 μL of Dimethyl sulfoxide, and the final volume reaction was 5 mL. The reaction was carried out overnight at RT under mild stirring. According to Scheme 1, panel B, the reaction involves the coupling of the isothiocyanate–N=C=S electrophilic group with the nucleophilic –OH of the polymer shell surface of the MBs by forming thiocarbamate groups [16]. The PVA MB dispersion was washed with MilliQ water with several steps of centrifugation (1000 rpm, 10 min each). DOTA functionalization was then evaluated from the difference between the DOTA feed amount added to the MB reaction mixture and the unreacted DOTA, by measuring the absorbance at 270 nm of the free chelator in the subnatant (Spark^®^, Tecan, and Corning UV transparent 96 well plates; linear p-SCN-Bn-DOTA concentration range 100 µM–14 µM), according to Equation (2) (see also Section 3.1). It is worth mentioning that the quantification of DOTA was indirectly assessed from the subnatant rather than from the functionalized MB suspension to minimize errors that arise from scattering.

(2) DOTA bound to MBs (n)=DOTA feed amount (n)−DOTA unreacted in subnatant (n)

The result was normalized by the MB number of the sample, as determined by optical microscopy, in order to obtain the molar amount of DOTA (n)/MB (Equation (3)):

(3) DOTA (n)MB=DOTA bound to MBs (n)number of MBs  

As a control check, in order to confirm that DOTA molecules were linked onto the PVA MBs by chemisorption, the same procedure of functionalization was performed with DOTA chelator being the isothiocyanate group previously deactivated in the protic solvent (i.e., overnight in MilliQ water). In this case, according to Equation (2), no moles of DOTA bound to MBs were detected.

Then, 2 × 10^8^ DOTA functionalized MBs were redispersed in 0.553 mL acetate buffer (0.1 M, pH 4.5), and 130 μg (0.67 μmol) of Yttrium chloride 10 mg/mL in acetate buffer was added to the PVA-DOTA MB suspension; the mixture was then incubated at 50 °C and stirred at 300 rpm for 1 h. After that, 0.566 mL acetate buffer 0.2 M, pH 5.6, was added and the free uncomplexed yttrium was removed by centrifugation at 1000 rpm for 5 min. The free yttrium amount was quantified on a known volume of the subnatant by direct complexometric titration using ethylenediaminetetraacetic acid (EDTA, Carlo Erba, Darmstadt, Germany) and Xylenol Orange (Sigma-Aldrich, Milano, Italy) as competing chelator and indicator [25], respectively, to determine by difference the loaded yttrium, and the loading efficiency of the method, normalizing to the MBs’ number (Equations (4) and (5)). In particular, the subnatant, 1 mL, was added with 6 µL of Xylenol Orange 0.1% in 1:1 (*v*/*v*) ethanol: water, then free yttrium was titrated with 5 mM EDTA aqueous solution (5 µL/injection). Finally, the end-point was taken when all reddishness disappeared and the solution turned yellow.

(4) Complexed Y (n)=Y feed amount (n)−uncomplexed Y in subnatant (n) 

(5) Yttrium loading efficiency (%)=Complexed Yttrium (n)MBDOTA (n)MB×100

For co-loading the PVA MBs with DOTA and cRGD (cRGD-PVA-DOTA MBs), 3 · 10^8^ cRGD-PVA MBs dispersed in 4.5 mL of MilliQ water were mixed with 1 mg of DOTA previously dissolved in 50 μL of DMSO. The functionalization was then carried out and quantified, following the same protocol reported above. The reaction was stopped by centrifuge rinsing 10 times with MilliQ water. The resulting cRGD-PVA-DOTA MB dispersion was stored at 4 °C. No significant difference was observed in DOTA loading efficiency on cRGD-PVA MBs with respect to that on PVA MBs. Notice that the co-loading procedure was also tested by reversing the derivatization order (i.e., by coupling cRGD onto PVA-DOTA MBs), resulting in a dramatic loss of cRGD loading efficiency.

### 2.4. Handling of Microcatheters with PVA MBs for Insertion in Microfluidic Channels

The in vitro compatibility experiments of PVA MBs in microcatheters were performed at 25 °C using the Magic 1.2 F catheter (Balt Extrusion, Montmorency, France) with 440 μm diameter distal shaft and the maximum operating pressure of 7 bar, without using the guiding catheter. Optically transparent microchannels (0.4 IbiTreat chambers, Ibidi, GE, and Vena8 Endothelial+Biochip, Cellix Ltd., Dublin, Ireland, with a depth of 400 μm or 100 μm, respectively) were employed to mimic the circulation of PVA MBs from the microcatheter into arteries and veins.

Briefly, the distal end of the catheter was inserted at the inlet of a microchannel already filled with sterile 0.9% NaCl saline. The mandrel of the catheter was used to assist the insertion of the distal end through the channel reservoir. Then, the mandrel was slowly removed to allow the connection of the proximal shaft end of the catheter with a 10 mL needle-free plastic syringe (Fisherbrand, Fisher Scientific, Rodano, Italy) already filled with PVA MBs in sterile normal saline dispersion (10^7^ MBs/mL in 0.9% NaCl). 

Preliminary injection tests operating the syringe manually were performed to verify the absence of obstructions causing excessive operating pressures, thus preventing any damage to the microcatheter and the PVA MBs. Thereafter, the injection syringe was mounted on a programmable, step-by-step motor-driven syringe pump system (New Era Pump Systems Inc., Brooklyn, NY, USA), which was set at different constant volumetric flow rates, ranging from 0.05 mL/min to 1.12 mL/min, and corresponding to shear rate values from 144 s^−1^ to 56,000 s^−1^ and shear stress from 1 dyne· cm^−2^ to 560 dynes ·cm^−2^, according to the microchannel supplier specifications [26,27].

The flux of MBs from the microcatheter through the microfluidic channel was monitored in real time via optical microscopy in a bright field and fluorescence modes using an inverted microscope (Nikon Inverted Microscope Eclipse Ti-E), equipped with an Andor Zyla camera (Zyla 4.2 sCMOS, Oxford, UK), together with a super high pressure mercury lamp (C-SHG1 Nikon) and a green He-Ne laser as light sources for fluorescence videos and CLSM imaging, respectively. A 40× long working distance objective (Nikon S Plan Fluor, Florence, Italy) was used, and the objective focal length was set near the top of the channel; then, images and videos of PVA MBs either floating up or moving along the channel were acquired and processed by NIS-Elements software (AR Ver 5.30.02, Nikon). Further images at the bottom of the channel were taken in order to also detect the possible presence of PVA MB capsules or shell debris formed by the rupture of PVA MBs. For fluorescence detection, RBITC MBs together with fluorescence Filter Cube for TRITC (tetramethyl-rhodamine isothiocyanate, excitation: 542 nm, Emission: 620 nm) were used.

### 2.5. Ultrasound Attenuation Spectroscopy of PVA MBs

MBs’ attenuation spectroscopy measurements were performed on plain and cRGD-functionalized PVA MBs using a customized experimental set-up according to [16]. In this configuration, the sample (about 17 cm^3^) was placed in a glass chamber (2 × 2 × 5 cm) under continuous stirring. The glass chamber had two opposite apertures, where the emitting and receiving transducers (Olympus, V311, Tokyo, Japan, 10 MHz) were in contact with the liquid. The distance between the two transducers was fixed and equal to 2 cm. The sample was irradiated by an incident ultrasonic signal in the form of consecutive sinusoidal bursts at varying frequency from 0.5 to 20 MHz (250 kHz steps), with a calibrated peak pressure that was never higher than 14 kPa. The ultrasound signal, attenuation spectra, and the phase velocity were controlled and acquired by a LabView-based (National Instruments Corporation, Austin, TX, USA) homemade software.

### 2.6. Adhesion of cRGD-PVA MBs Carriers onto Endothelial Cell Channels

The channels (μ-Slide I 0.4, Ibidi, Gräfelfing, Germany) for the adhesion experiments of cRGD-PVA MBs, during their flow onto human umbilical vein endothelial cells (HUVECs), were prepared following the protocol reported in the literature [28], which is briefly described below.

Each channel was filled with a 2% bovine gelatin (Sigma-Aldrich, Milano, Italy) solution and left to dry for 2 h, followed by 10 washes with sterile PBS. The gelatin present on the internal walls of the microchannel promotes adhesion and cell growth. For a typical experiment, about 10^5^ HUVEC cells (Promocell), grown with the supplemented endothelial cell medium in a humidified cell incubator (37 °C, 5% CO_2_/air) [28], were inserted into the channel, which was then immediately turned upside-down and left overnight in the incubator to allow the cells to adhere to the upper wall of the channel. These growth conditions, considered to favor the adhesion of MBs to the cells, deploy the floating tendency of MBs in aqueous media. The following day, three washes of the channel using the culture medium, previously kept at 37 °C, were carried out to eliminate the residues of cells that did not adhere to the surface of the channel.

To carry out the adhesion experiments, the channel, in which the HUVECs were adhered, was placed under the inverted microscope (Nikon Eclipse Ti-E, 40× long distance objective). Through silicone tubes (internal diameter 1.6 mm, external diameter 3.2 mm), the channel was connected to a 10 mL needle-free plastic syringe filled with a suspension of MBs in PBS, 10^7^ MBs/mL added with 10% *v*/*v* of basal grow culture medium (Promocell, Heidelberg, Germany). The channel prepared as previously described was placed in a mini-incubator (Ibidi temperature controller ibiTC-288), which was necessary to carry out the observations at 37 °C, without stressing the layer of cells adhered to the channel walls.

The sample containing functionalized MBs was fluxed into the channel with a flow rate of 1.12 mL/min, which in the μ-Slide I 0.4 channel corresponds to a shear stress of 1 dyne/cm^2^ and is close to the values accomplished by the blood stream in postcapillary vessels.

The expression of α_v_β_3_ integrins on the used HUVEC cell line was quantitatively verified by immunofluorescence method (see Section 1 of ESI for details).

## 3. Results

In this section, we first highlight the formulation of echogenic PVA-based MBs as a suitable microdevice for loading and transporting radioactive Y cargo and targeting tumor-associated endothelial cells via integrin recognition. Then, we report on PVA MBs’ suitability for in situ injection via microcatheter and the accumulation of targeted PVA MBs on the HUVEC cell line as a model of integrin overexpression.

### 3.1. Characterization of Engineered PVA MBs for Yttrium Transport

**Figure 1:**
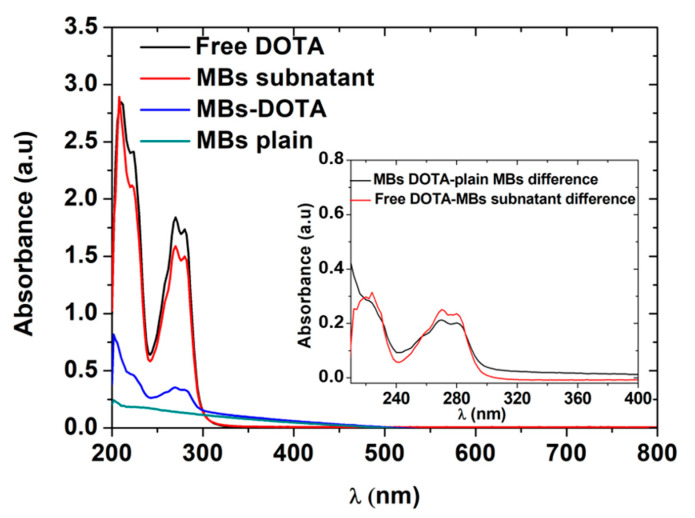
UV-Vis absorption spectra of DOTA (0.18 mM) (black line) removed DOTA excess in MBs’ subnatant (red line), PVA-DOTA MBs (blue line), and plain PVA MBs (cyan). The inset is the absorption spectrum of PVA-DOTA MBs after scattering correction (black line), and the difference between free DOTA and unbound DOTA in the subnatant of the functionalized MBs (red line).

DOTA bifunctional chelator used herein, p-SCN-Bn-DOTA, is known for its ability to cage Indium (III) and lanthanide ions such as Y(III). DOTA scaffold features a distinctive absorbance band at 270 nm attributed to the aromatic moiety (see also Scheme 1, panel B). It is worth noting that this absorption is maintained after the conjugation of DOTA with the PVA shell, whereas in the UV absorption spectrum of the plain MBs, the 270 nm absorption is absent (see Figure 1). Additionally, the absorbance value of the functionalized MBs at 270 nm is consistent with the difference between the initial amount of DOTA and the unreacted chelator present in the subnatant after its reaction with the MBs (see the inset of Figure 1). Therefore, on this basis, we can assess the number of DOTA molecules coupled to the MBs’ surface as (1.0 ± 0.2) × 10^9^ molecules of DOTA/MB (~2 × 10^−15^ mol/MB). 

Notice that the details concerning the formulation of cRGD-PVA-DOTA MBs are reported in Materials and Methods and in Section 2 of ESI where the calibration curve of cRGD (Figure S2) and representative absorbance spectra of DOTA (Figure S3) are also shown. It resulted in a loading efficiency of (1.0 ± 0.2) × 10^8^ cRGD/MB and (1.1 ± 0.3) × 10^9^ DOTA/MB.

DOTA chelates Yttrium in a 1:1 stoichiometric ratio and the resulting octadentate DOTA complexes exhibit high thermodynamic stability (pK ≈ 24.3 at 25 °C) [29], allowing the use of their derivatives for medical purposes [30]. According to the reported radiolabeling protocols, DOTA optimal Y chelating conditions are pH 4.5–6 at 50 °C [30]. DOTA functionalized MBs were incubated with a two-fold molar excess of Yttrium chloride (YCl_3_) over the moles of DOTA coupled with the MBs sample. The loading reaction was carried out for 1 h at 50 °C, then the direct titration of uncomplexed free Yttrium yielded a loading of (5.0 ± 0.3) × 10^−16^ moles of Yttrium/MB, corresponding to about 25% of DOTA on MBs complexed with Yttrium. 

Notice that the loading and quantification of Y on cRGD-PVA-DOTA MBs were performed in duplicate following the same protocol described for PVA-DOTA MBs. It led to an average value of (5.2 ± 0.3) × 10^−16^, indicating that the Y loading efficiency is not affected by the presence of cRGD on the surface of the PVA-DOTA MBs carrier.

### 3.2. The MBs’ Response to Ultrasound

**Figure 2:**
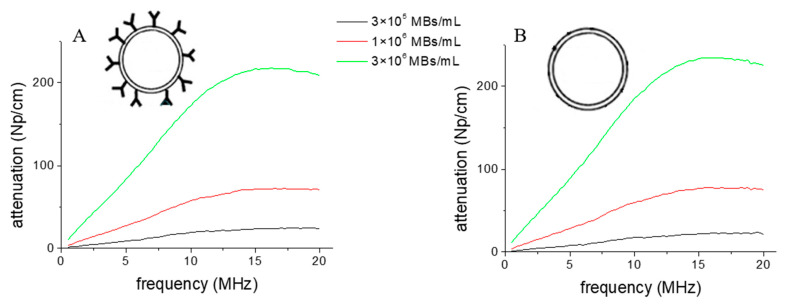
The attenuation coefficient at varying ultrasound frequencies performed on PVA MBs completely covered with cRGD (**A**) and naked PVA MBs (**B**) are compared at three different number densities in water dispersion (3·10^6^, 1·10^6^, 1·10^5^ MBs/mL as specified in the graph label). At a fixed number density, the shapes of the corresponding attenuation profiles are identical, with the maximum at the same acoustic resonance frequency of 13.5 MHz.

In Figure 2, the comparative ultrasound attenuation spectra at different concentrations of plain and functionalized MBs are shown. The spectra of functionalized MBs correspond to the fully capped MBs (i.e., all carbonyl groups of the shell have been replaced), with the cyclic RGD peptide representing the most perturbative derivatization of the plain MBs’ shell. As expected, this MBs’ functionalization procedure does not affect the shape and the intensity of the attenuation profile of PVA MBs, whose resonance frequency remains centered at 13.5 MHz according to literature on plain PVA MBs [16]. In fact, according to Scheme 1, the modification of the surface of the MBs (analogously for linking DOTA or cyclic RGD) consists of the covalent coupling of small ligands (MW ~600 Dalton) over several nanometers’ length of telechelic PVA protrusions [15], at the interface with the aqueous medium. Therefore, we do not perturb the crosslinked PVA backbone composing the MBs’ shell, the latter conferring the shear modulus and the echogenic properties of the MBs. It is also worth noting that according to literature, such PVA-based elastomeric MBs exhibit a stiffness comparable to that of highly echogenic lipid MBs (i.e., 16.7 N/m and 14.1 N/m, respectively) [16].

For reasons of completeness, we also analyzed the echogenic response of the carrier, cRGD-PVA-DOTA MBs, in terms of ultrasound extinction cross-section after the carrier passed through the neurointerventional catheter. The analysis was reported in detail in Section 2 of ESI (Figure S4 and Table S1) and pointed out that the cRGD-PVA-DOTA MBs remain echogenic, exhibiting resonance frequency (13.5 ± 0.2 MHz) and acoustic damping coefficient (2.0 ± 0.1) values both consistent with the ones reported in the literature [15,16] for naked, unperturbed PVA MBs.

### 3.3. Microcatheter Compatibility Experiments

**Scheme 2:**
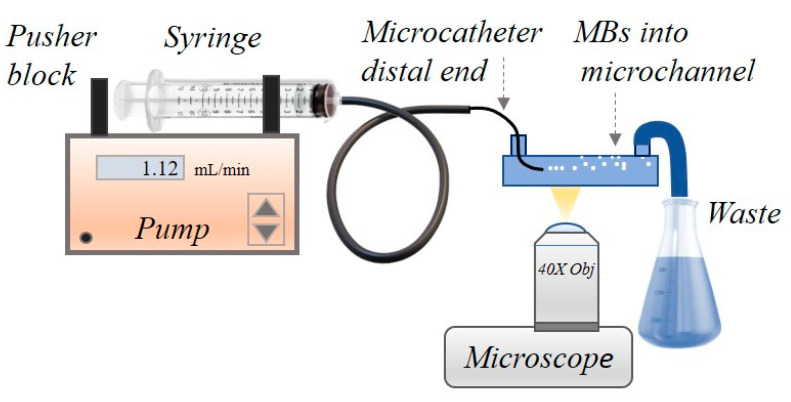
Sketch of the setup for microcatheter infusion and real-time imaging of the PVA MB dispersion in microfluidic channel.

**Figure 3:**
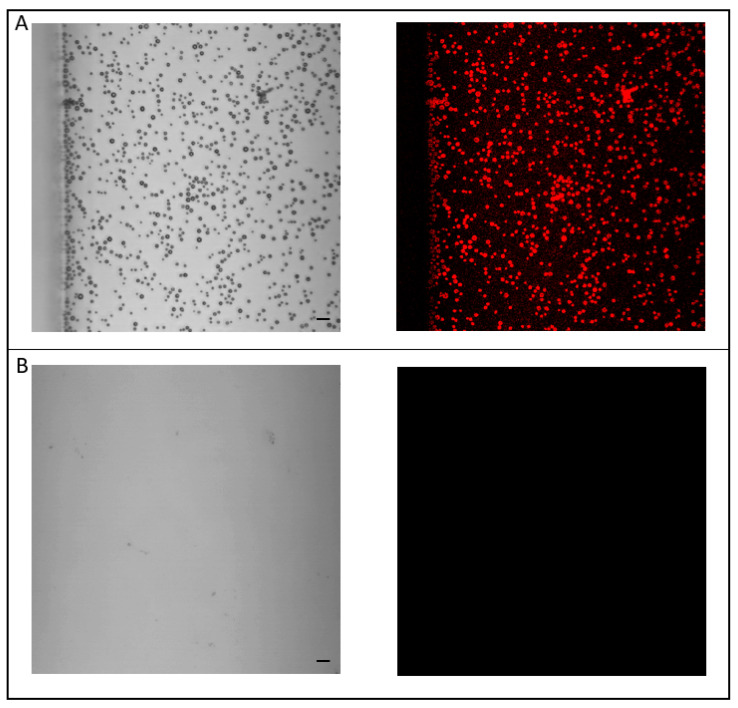
Bright field (**left**) and CLSM images (**right**) of (**A**) RBITC MBs into the microchannel Vena8 Endothelial + (the edge of the channel is visible on the left side); (**B**) images at the bottom of the same channel showing the lack of capsules and debris from RBITC MBs. Scale bar 10 μm.

Video microscopy (Video ESI) and micrograph images (Figure 3) are representative of the PVA MB dispersion poured into a fluid channel through the microcatheter, according to the flow system setup sketched in Scheme 2.

The videos show that PVA MBs can flow without hindrances and concentration gradients within the microchannel over a wide range of shear rates. According to Figure 3, panel A, the MBs seem not to undergo alterations to their native morphological and dimensional features (i.e., spherical shape with the size of 4.3 ± 0.7 μm, according to the CLSM characterization reported in Section 2. Further confirmation is given by the absence of capsules and debris that may arise from the damage to the polymeric shell of the MBs that underwent significant shear stress (Figure 3, panel B). Notice that the latter observation also applies successfully to the MBs functionalized with cRGD and DOTA and injected via the catheter into the channel covered with HUVEC cells. In fact, the cRGD-PVA-DOTA MBs that have passed through the catheter maintain the echogenic response of the unperturbed PVA MBs [15,16] (see also Section 2 of ESI, Figure S4), as well as the targeting ability provided by the cRGD ligand (as shown in the next sections).

This means that the microcatheter is compatible with the injection of the PVA MBs, also when formulated as targeted Y-carrier, in the microfluidic conditions of shear stress, pH, and temperature, resembling those occurring in the vascular system of small animals with vessel sizes of about 100 and 400 μm, and shear stress ranging from 1 dyne·cm^−2^ to 560 dynes cm^−2^ (e.g., artery of a mouse, blood vessels of rats, etc.) [31].

As shown in the next section, the flow chamber devices were also exploited to provide information about the interaction between endothelial cells and the PVA MBs carrier, under the flow condition of mechanical stimulation (i.e., shear stress of 1 dyne/cm^2^), where the endothelial cells in postcapillary vessels are constantly exposed, and through which vascular tone and homeostasis is regulated [28,32].

### 3.4. Endothelial Cell Targeting of cRGD-PVA MBs

The in vitro adhesion tests are necessary to gather quantitative information concerning the active targeting and efficiency of the selective accumulation of cRGD-PVA MBs through the interaction between cRGD-lys and integrin α_v_β_3_. The targeting experiments were performed on the HUVEC cell line, which is characterized by a high expression of integrin α_v_β_3_. For this peculiarity, they are currently used for the development of vascularized in vitro tumor models to study GBM angiogenesis and cancer invasion, in which endothelial cells are reprogrammed to increase the expression of integrins α_v_β_3_ [24].

**Figure 4:**
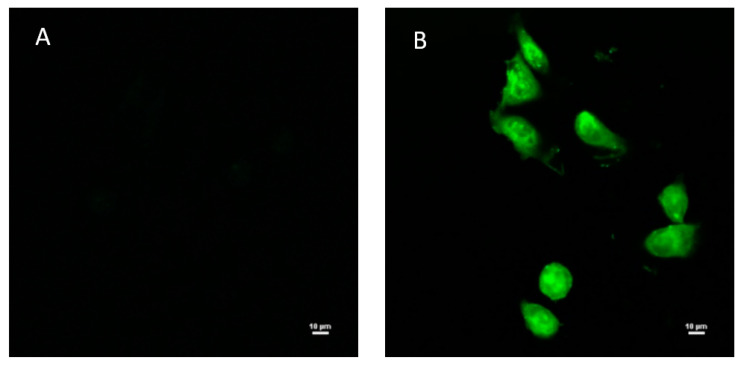
Fluorescent images of HUVEC cells in the absence of Ab (**A**) and titrated with a solution of green fluorescent Ab 5 μg/mL (**B**) revealing the presence of a high density of integrins α_v_β_3_. Scale bar: 10 μm.

An immunofluorescence assay was used for the assessment of the integrin α_v_β_3_ surface density of the HUVEC cell culture exploited in the targeting experiments (for details, see Section 1 of ESI). The representative fluorescence images are shown in Figure 4, and the relative quantitative determination is provided in Figure S1 of ESI. The results confirm the high expression of integrins α_v_β_3_ in HUVEC cell cultures, which we estimate to be around 90,000 receptors/cell, in accordance with the reference value of 10^5^ receptors/cell measured on the HUVEC by flow cytometry [33]. 

**Figure 5:**
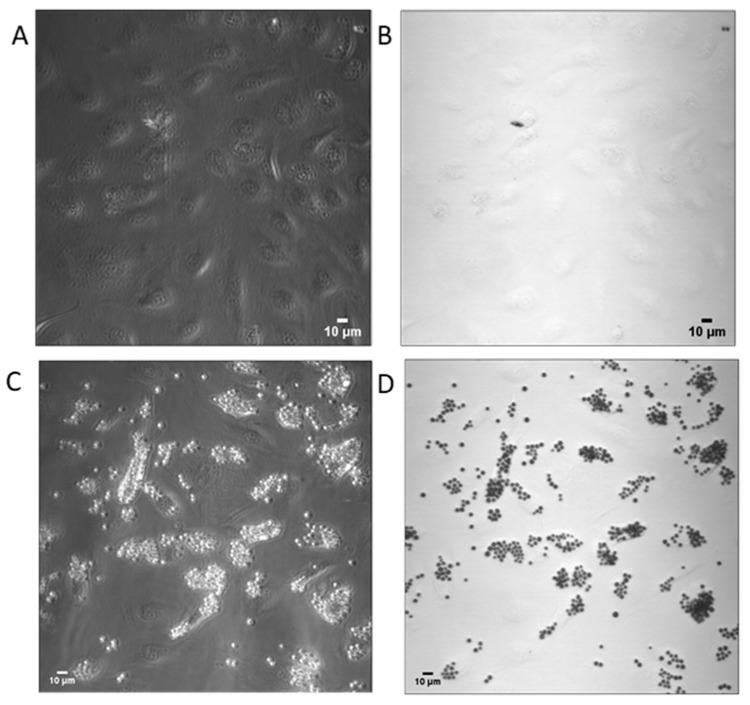
Optical microscopy images (long distance objective 40×) of targeted PVA-cRGD MBs’ adhesion on HUVEC cells in a dynamic flow at 37 °C post-fluxing (10 min), inversion (5 min), and washing with PBS (10 min), in comparison with plain MBs: (**A**,**B**) cell interaction with plain PVA MBs (control) in phase contrast and bright field, respectively; (**C**,**D**) cell interaction with PVA-cRGD MBs and phase contrast and bright field, respectively.

Suspensions of cRGD-PVA MBs at a concentration of 10^7^ MBs/mL were fluxed inside the channels on which the HUVECs were grown. The studies were conducted in a 333 × 333 μm^2^ sized channel section using a 40× long-distance objective. After 10 min of flowing cRGD-modified MBs, the HUVEC channel with adhered cRGD-PVA MBs was turned upside-down and washed with a solution of PBS added with a 10% basal medium to exclude the presence of any weak and non-specific interactions between the MBs and the cell substrate. Figure 5 shows the representative images recorded after the MBs’ perfusion and washing, indicating a significant accumulation of cRGD-PVA MBs onto the HUVEC cells (Figure 5C,D). The result suggests a good affinity between cRGD-PVA MBs and HUVEC cells overexpressing α_v_β_3_ integrins in the used flow conditions of 90,000 receptors/cell. No significant event of interaction between the bare PVA MBs and the HUVECs was detected (Figure 5A,B), indicating an accumulation of the PVA MBs on the cell surface driven by specific recognition between cRGD-MBs and the integrins α_v_β_3_.

Based on these in vitro results, we estimated the number of bubbles per cell surface at 12 ± 4, which are potentially capable of localizing at the vascularized tumor sites.

### 3.5. Endothelial Cell Targeting of cRGD-PVA-DOTA MBs

**Figure 6:**
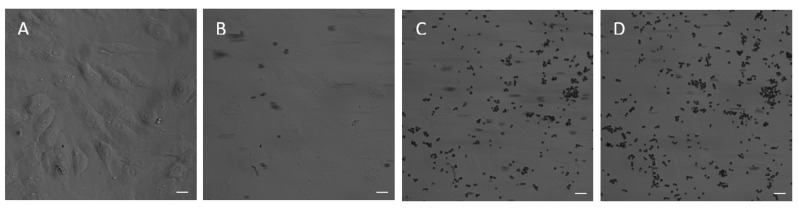
Optical microscopy real-time images (long distance objective 40×) of the PVA MBs carrier co-loaded with DOTA and cRGD (cRGD-PVA-DOTA MBs) targeted on the α_V_β_3_ integrins of HUVEC cells (frame (**A**) a control image of the top side of the HUVEC-coated fluid channel), acquired under dynamic flow at 37 °C, shear rate 147 s^−1^ (shear stress 1 dyne/cm^2^); frame (**B**) the carrier was just injected in the channel (t = 0 min); frame (**C**) after 5 min of fluxing the carrier; frame (**D**) after 10 mi of fluxing (10 min). The control micrograph image shown in panel A was acquired in phase contrast mode to highlight the cell substrate; afterwards, on the same frame, the acquisition of the time-lapse sequence shown in panels (**B**–**D**) was set in bright field, focusing on the adhered MBs’ plane, to optimize the MBs’ visualization and processing by NIS-Elements software (AR Ver 5.30.02, Nikon). Scale bar: 20 μm. Upon washing the channel with PBS solution mixed with 10% of bovine serum, the carrier remains adhered onto the HUVEC cells substrate.

After successfully demonstrating that by linking cRGD on the surface of PVA MBs, it is possible to concentrate PVA MBs on the endothelial cells overexpressing α_v_β_3_ integrins, we also verified that the Y-carrier, cRGD-PVA-DOTA MBs, actually maintains the same ability. The results are shown in Figure 6 (see Section 2 of ESI for experimental details). 

Specifically, Figure 6, panel A, shows the image of the HUVEC cell line adherent to the upper wall of the microchannel, obtained through the 40× long distance objective, in phase contrast mode, by an inverted Nikon Eclipse Ti-E microscope. To carry out the in vitro adhesion experiments, the set-up shown in Scheme 2 was used. A total amount of 10^7^ cRGD-PVA-DOTA MBs/mL in PBS containing 10% *v*/*v* basal culture medium were injected within the HUVEC-coated channel via catheter in physiological conditions, under stationary flow resembling an authentic endothelial vasculature shear stress of 1 dyne/cm^2^. The use of a mini-incubator (Ibidi temperature controller ibiTC-288) allowed the experiments to be carried out at 37 °C to guarantee the best physical conditions. 

Figure 6, panels B, C and D, shows representative micrograph images of the top side of the HUVEC-coated channel, which we captured during the successful progressive adhesion of cRGD-PVA-DOTA MBs, monitored in real-time for 10 min. We did not observe significant differences with respect to the binding ability of the MBs loaded with cRGD only on HUVECs. Bearing in mind that the value of Y moles per MB that can be loaded on cRGD-PVA-DOTA MBs is (5.2 ± 0.3) × 10^−16^ (which is equal within error to the ones obtained for PVA-DOTA MBs), the amount of Y per cell transported by this carrier upon injection via microcatheter onto the HUVEC cells can be calculated according to Equation (6):

(6) Delivered Yttrium (n)cell=(Complexed Yttrium (n)MB)×(n° adhered MBscell)

We estimated (4 ± 1) × 10^9^ molecules/cell, corresponding to an average value of ~13 ± 4 MBs adhered on a HUVEC cell.

## 4. Discussion

### 4.1. Theranostic MBs

The recent physico-chemical advances of polymer ultrasound contrast agents are transforming the concept of MBs into invaluable new multi-responsive platforms of theranostic relevance, combining early diagnosis and therapeutics [34,35,36]. It has been demonstrated that the crosslinked PVA chains of the shell behave as a biocompatible elastomeric three-dimensional network, providing high structural stability, even in quite acid tumor-like environments, and relevant echogenicity [16,37]. It is also noteworthy that the ease of derivatization of PVA allows for targeted, tunable strategies of drug-delivery [15,38,39]. 

For this purpose, we herein demonstrated that both hydroxyl and aldehyde groups of the oxidized PVA network can be easily exploited to chemisorb at the MBs’ surface an Yttrium chelating complex (~10^9^ DOTA molecules/MB) and a tumor-targeting ligand cRGD (~10^8^ molecules/MB), respectively. More specifically, PVA MBs can be engineered with a derivative of DOTA chelator to include Yttrium at the water interface of the PVA MBs, which behave thus functionally as a Y90 carrier to be directly introduced via catheter into the GBM-associated vasculature for targeted radiotherapy. Furthermore, the modification of PVA MBs’ surface with DOTA, and its ability to chelate Yttrium, opens the possibility to expand the use of MBs beyond ultrasound, as Y86 tracers for positron emission tomography (PET) imaging and as a contrast agent for hyperpolarized magnetic resonance imaging with the natural isotope Y89 [30]. 

As mentioned in the introduction, MBs move through the vascular network without extravasating. The demonstrated possibility of functionalizing PVA MBs with cRGD, also in co-loading with DOTA, paves the way for new strategies in which the super-selectivity of the microcatheter could be combined with the targeting induced by the cRGD—α_v_β_3_ integrins biorecognition on the tumor endothelium. This would allow the Y-carrier to accumulate on the tumor endothelium to activate molecular imaging and radiotherapy with the hope of implementing the radioembolization therapy to maximize the collateral benefit/risk ratio.

### 4.2. Injections Compatibility via the Neurointerventional Catheter

We have shown that this potential Y90 carrier is compatible with injections via a neurointerventional catheter (i.e., 1.2F). Our in vitro experiments have demonstrated a very low coefficient of PVA MB friction and aggregation, which translated into two highly desirable ramifications: first, the absence of particle “stick” within the microcatheter, without any hindrance or increase in the resistance during the injection; second, and probably the most worthy, is that we observed the full control of the PVA MBs’ insertion rate, which includes the ability to reverse the MBs’ flow without causing obstruction, even in the case of back-flow induced in the microcatheter. Moreover, no damage to the carrier was observed after the forward and backward injection maneuvers, as well as to the PVA MBs previously linked with cRGD and DOTA and injected via catheter for the cell adhesion experiments herein reported. In our opinion, this result should deserve particular attention since the microcatheter used had an outer diameter of 1.2F, and it is the smallest flow-directed microcatheter dedicated to neuroendovascular procedures available at the moment on the market. The occurrence of the particles’ aggregation during embolization is not uncommon and they clump together with the microcatheter occlusion [40]. This eventuality is even more frequent when conventional microcatheters smaller than 2.4F are used. In this situation, the forceful injection in an effort to clear the system may result in a significant reflux of the embolization agent in nontargeted areas, potentially resulting in devastating consequences if the embolization is performed in the brain [41]. The demonstration of MBs’ compatibility and the ability to perform different successful injection maneuvers provide a step forward, with the use of the current neurointerventional materials, to target brain tumoral lesions. 

### 4.3. Adhesion Experiments

The adhesion experiments of cRGD-PVA MBs, also co-loaded with the chelator of Y, DOTA, have shown the concrete possibility of accumulating Y90 carriers in a vascular network, herein simulated by HUVEC cell channels overexpressing integrins α_v_β_3_, the latter being a well-known molecular target of the vascular endothelium of GBM. The detail of the interaction between MBs and HUVECs in steady flow is the subject of a dedicated study. Herein, the rationale of the targeting test has been to prove that the tumor can be efficiently targeted via cRGD-coupled MBs, and that this will yield a delivery of about 6 × 10^−15^ moles of Y90 per HUVEC cell, through the cell-adhered microcarriers.

We would also discuss additional, peculiar vantages in choosing MBs with a robust elastomeric shell as a scaffold of the targeted microcarriers. Among them, it is worth noting that, in comparison to the nano- or micro-particles with a solid- or liquid-core, the air-filled PVA MBs feature much lower mass, while maintaining a large biocompatible surface. On such a surface multiple pharmaceutics, ligands, and tracking molecules (e.g., via NMR and microfluorescence) [15], coexisting in a single MB overall can interface on the tumor cells. This may result in a satisfactory loading capacity and provide a low impact on biodistribution and biodegradation of the carrier amount. Secondly, PVA MBs can be solicited through the medical ultrasound stimuli to cavitate and enhance plasma membrane sonoporation, blood-brain barrier (BBB) permeabilization, and blood vessel extravasation of pharmaceutics (e.g., facilitating therapeutics delivery through the abnormal tumor-associated endothelial cells network) [42], whose effectiveness has been studied by preclinical and clinical trials [43,44]. In particular, in a pilot in-humans study [44], it has been demonstrated that US-triggered microbubble destruction and transarterial radioembolization can be combined to figure out a feasible and safe protocol, leading to an improvement in the response to treatments in hepatocellular carcinoma.

In this way, proceeding towards personalized medicine approaches, we wonder if in combination with the ultrasound stimuli, the PVA MBs could earn clinical interest as Y90 carriers in maximizing the cytotoxic effects of Y-based radiotherapy through locoregional infusion, while also helping to (i) visualize and circumscribe GBM tumor portions and also (ii) support ultrasound-enhanced concomitant targeted chemotherapy.

### 4.4. A New Radiotherapeutic Frontier for the Treatment of Radioresistant GBM

Despite major advances in radiation technology, the overall outcome of radiotherapy in GBM remains far from optimal, as GBM recurrences are inherently radioresistant. Given the lack of effective pharmacological and surgical treatments for GBM recurrences, its speed of growth, and its heterogenous cytological nature, a novel treatment modality has the potential to be a game changer in terms of the patient’s survival. The radiotherapy challenges, including tumor heterogeneity, and metabolic alterations make it difficult to cure GBM recurrences using conventional approaches [45,46]. The combination of MBs and the minimally invasive super-selective endovascular radiopharmaceutical infusion of ionizing radiation may limit the off-target toxicities to the normal brain, minimizing non-target radiation with an average tissue penetration of 4.1 mm as opposed to 3–4 cm in typical I-125 brachytherapy or External Beam Radiotherapy, where the entire cortex may be exposed to substantial doses of radiation.

## 5. Conclusions

Our results show that the PVA MBs easily implemented for bearing DOTA and cRGD on the shell surface become an efficient Y-carrier that exhibits selective affinity for the cell surfaces that overexpress α_v_β_3_ integrins (that is for the HUVEC cell line). Moreover, this carrier is echogenic and compatible with the use of low-sized catheters. In this way, the MBs can be used as a carrier to concentrate radioactive lanthanides such as Y90 on such cell surfaces, through a neurointerventional catheter injection.

Since integrins α_v_β_3_ represent an important target for imaging and treating gliomas, as well as perturbing tumor-induced angiogenesis, we are confident that this system may provide an encouraging basis for in vivo investigations to evaluate the combined use of neurointerventional catheters and cRGD-targeted MBs for designing a minimally invasive super-selective intra-arterial approach against GBM recurrences. In vivo evidence on the efficacy of this Y90-carrier against glioblastoma will be presented in a forthcoming paper.

Even more generally, we hope our approach may impact the development of more precise and personalized medicine, as well as new medical devices. 

## Data Availability

Data is contained within the article or supplementary material.

## Supplementary Materials

The following supporting information can be downloaded at https://www.mdpi.com/article/10.3390/pharmaceutics15010217/s1, Figure S1: On the left is the calibration curve of the free Ab-Alexa Fluor in PBS. On the right is the plot of immunofluorescence titration of HUVEC α_V_β_3_ integrins using Ab; Figure S2: Fluorescence calibration curve of cRGD provided from phenylalanine (f) emission as measured in triplicate by Spark multimode microplate reader (TECAN). Ex. λ = 235 nm; Em. λ = 283 nm; bandwidth 20 nm; Figure S3: Representative UV absorbance spectra of DOTA in water solution at the initial concentration of 0.32 mM (black line), and the removed DOTA excess (0.20 mM) in the cRGD-PVA-DOTA MBs’ subnatant (red line); The solutions were diluted 1:5 in MilliQ water before acquisition as measured in triplicate using a Spark Tecan multi-well plate reader. From the absorbance values at 270 nm, we pointed out 1.1×10^9^ DOTA/MB (see also Materials and Methods, Equation (3)); Figure S4: Graph showing the extinction cross-section profile as a function of ultrasound frequency averaged from the attenuation profiles measured at (0.5, 1.0, 2.0, 2.5)×10^6^ MBs/ml; the best fit profile (superimposed in red line) according to Equation (S1) is adherent to σ_e_ in the relevant frequencies’ subrange of 6.5-20 MHz; Table S1: Resonance frequency and viscous damping coefficient values of cRGD-PVA-DOTA MBs in water dispersion that have passed through the neurointerventional catheter, as fitted according to the best-fit model (Equation (S1)); Video S1: injection of PVA MBs via microcatheter in microfluid channel.
