# Supplementary material for: PVA-Microbubbles as a Radioembolization Platform: Formulation and the In Vitro Proof of Concept
Source: Pharmaceutics. 2023 Jan 8;15(1):217. doi: 10.3390/pharmaceutics15010217 (PMC9862136; doi:10.3390/pharmaceutics15010217)
Supplement: Supplementary file 1 [file pharmaceutics-15-00217-s001.zip › pharmaceutics-2012935-supplementary.pdf]

PVA-microbubbles as a Radioembolization Platform: Formulation and the *in vitro* Proof of Concept

Electronic Supporting Information

Valerio Da Ros[1], Letizia Oddo[2], Yosra Toumia[2], Eugenia Guida[1], Silvia Minosse[3], Lidia Strigari[4], Silvia Strolin[4], Giulia Paolani[4], Francesca Di Giuliano[1], Roberto Floris[1], Francesco Garaci[1], Susanna Dolci[1], Gaio Paradossi[2], Fabio Domenici[2,*]

[1] Department of Biomedicine and Prevention, University Hospital of Rome "Tor Vergata", 00133 Rome, Italy

[2] Department of Chemical Science and Technologies, University of Rome "Tor Vergata", 00133 Rome, Italy

[3] UOC Diagnostica per Immagini, University Hospital of Rome "Tor Vergata", 00133 Rome, Italy

[4] Department of Medical Physics, IRCCS Azienda Ospedaliero-Universitaria di Bologna, 40138 Bologna, Italy

*Corresponding author; email address: fabio.domenici@uniroma2.it

S1. Integrins $\alpha_v\beta_3$ quantitation on HUVEC cells

The determination of integrins $\alpha_v\beta_3$ on HUVEC was performed by immunofluorescence method. About 10000 cells in the supplemented endothelial cell growth medium were seeded inside 8 different wells of a 96-well microplate, and cultured for 24h in a cell incubator at 37 °C in a CO_2-air humidified atmosphere of 5%.

Thereafter, the cells were fixed with cold (4 °C) 4% paraformaldehyde solution in PBS for 15 min. Cells were then washed three times with PBS and incubated with Ab-Alexa Fluor solutions at increasing concentrations (0, 0.1, 0.5, 1, 2, 3, 4, 5 μg / ml) in dark conditions at room temperature. After 3 h of incubation, the cells were washed 4 times with PBS to remove any unbound Ab-Alexa Fluor molecule and the microplate was inserted in a plate reader (Tecan SparkControl). The fluorescence signal was collected at the emission wavelength of 520 nm by irradiating the cells at the excitation wavelength of 470 nm. The fluorescence measurements were carried out as a function of the concentration of Ab-Alexa Fluor incubation solutions and compared with a calibration curve reporting the fluorescence intensity of the free Ab-Alexa Fluor as a function of the concentration in PBS (concentration range 0 - 0.5 μg/mL). Integrins surface densities normalized for the number of cells were derived from the calibration measurements on Ab-Alexa Fluor solutions, shown in **Figure S1**. The saturation point at 5 μg / ml was used to determine the amount of Ab necessary to bind all the receptors sites and corresponds to an integrins surface density of about 90,000 receptors/cell.

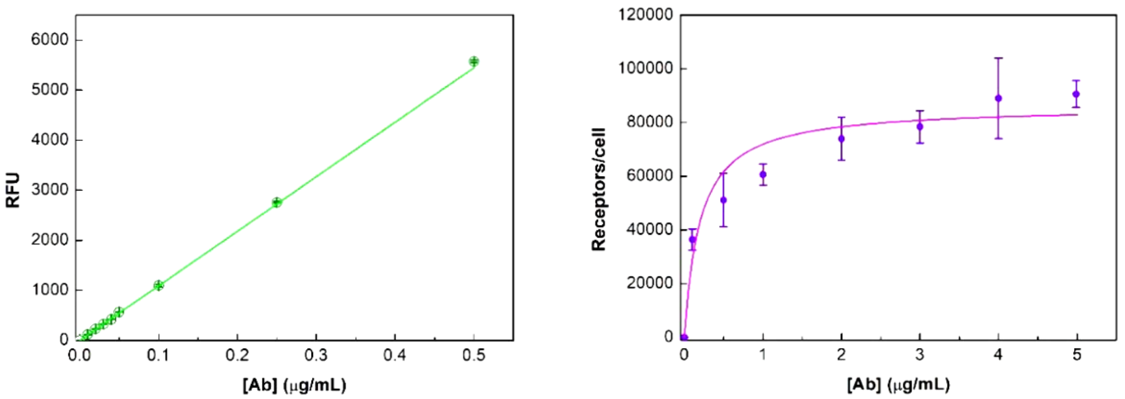

Figure S1. On the left is the calibration curve of the free Ab-Alexa Fluor in PBS. On the right is the plot of immunofluorescence titration of HUVEC $\alpha_V\beta_3$ integrins using Ab.

Microfluorescence of HUVEC cells whose integrins $\alpha_v\beta_3$ were saturated with 5 µg / ml of free Ab-Alexa Fluor addition was performed using a Nikon Inverted Microscope Eclipse Ti-E equipped with a super high-pressure mercury lamp (C-SHG1 Nikon) as light source, a FITC fluorescence filter block (dichroic filter from 500 nm; excitation filter 450nm/490nm, emission from 515 nm, Chroma Technology Corp), sCMOS camera (Zyla 4.2, Andor, UK), and a Nikon Plan Apo 60XA/1.40 Oil DIC H WD 0.21 objective, and ultrafast shutter (Lambda SC controller, Shutter Instrument). For image acquisition and elaboration, the software NIS-Elements AR, version 4.3 (Nikon) was used.

A representative fluorescence image corresponding to 5 µg / ml Ab-Alexa Fluor addition is shown in Figure 4 of the main text, which confirms the presence of a high density of integrins $\alpha_v\beta_3$ on the HUVEC cell membranes.

S2. cRGD-PVA-DOTA MBs

Formulation details. As reported in the Materials and Methods of the manuscript, PVA MBs were previously linked with cRGD exploiting the carbonyl groups of the oxidized PVA chains at the water interface. After the reaction, the fluorescence signal of the free cRGD from the water waste of the first centrifuge washing (i.e., the signal from the unbound cRGD with respect to that of the overall cRGD concentration), was analyzed through the calibration curve shown in **Figure S2**. In this way, we estimated $(1.0 \pm 0.2)\times 10^8$ of cRGD per MB.

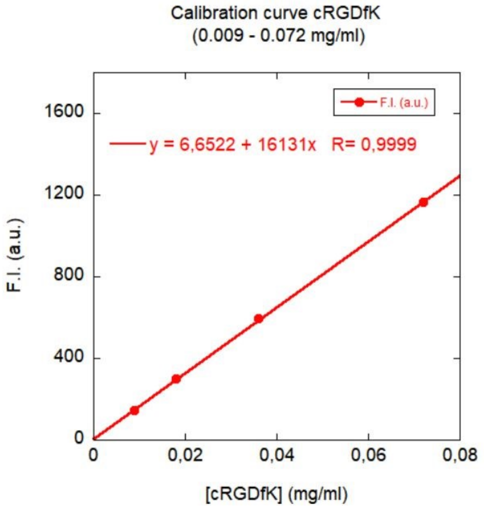

Figure S2. Fluorescence calibration curve of cRGD provided from phenylalanine (f) emission as measured in triplicate by Spark multimode microplate reader (TECAN). Ex. λ = 235 nm; Em. λ = 283 nm; bandwidth 20 nm.

cRGD-PVA MBs were in turn bound with DOTA by OH groups of PVA, resulting in $(1.1 \pm 0.3)\times10^9$ DOTA per MB according to absorbance spectroscopy performed from two independent preparations. Representative absorbance spectra of DOTA water solution, diluted of a factor 5, and measured before (Free DOTA) and after the reaction (MBs subnatant) are superimposed in **Figure S3** (for details of the quantification procedure see also Materials and Methods of the main manuscript together with the representative spectra shown in Figure 1).

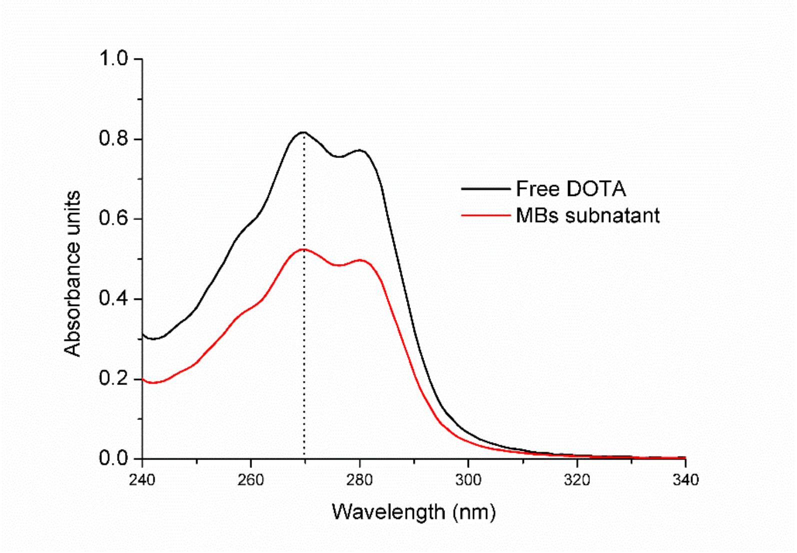

Figure S3. Representative UV absorbance spectra of DOTA in water solution at the initial concentration of 0.32 mM (black line), and the removed DOTA excess (0.20 mM) in the cRGD-PVA-DOTA MBs' subnatant (red line); The solutions were diluted 1:5 in MilliQ water before acquisition as measured in triplicate using a Spark Tecan multi-well plate reader. From the absorbance values at 270 nm, we pointed out 1.1×10^9 DOTA/MB (see also Materials and Methods, Equation (3)).

Echogenicity. For acoustic attenuation measurements, 5 ml of 10^7 MBs/ml cRGD-PVA-DOTA MBs in MilliQ water dispersion was manually injected with a syringe into the radiointerventional catheter. Subsequently, the dispersion recovered in a vial at the catheter outlet was diluted and measured using the experimental setup described in Materials and Methods of the main manuscript.

Specifically, the acoustic extinction cross-section was found by normalizing the acoustic attenuation profiles of four cRGD-PVA-DOTA MBs water dispersions $(0.5, 1.0, 2.0, 2.5)\times10^6$ MBs/ml for the respective number density, then averaging the results. The resulting plot together with the best fit is

shown in **Figure S4**. The quite small standard deviation of the average curve reflects the satisfying linearity and repeatability of the measurements at varying the MBs number density.

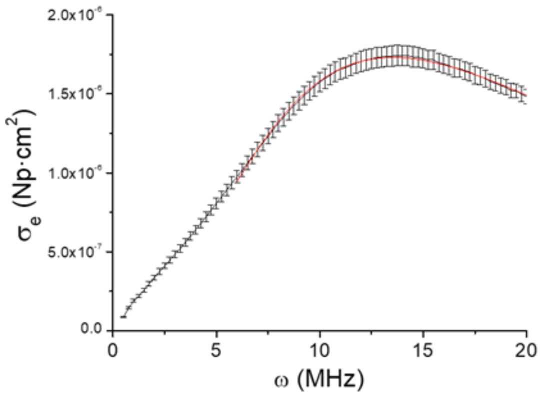

Figure S4. Graph showing the extinction cross-section profile as a function of ultrasound frequency averaged from the attenuation profiles measured at (0.5, 1.0, 2.0, 2.5)×10^6 MBs/ml; the best fit profile (superimposed in red line) according to Equation (S1) is adherent to σ_e in the relevant frequencies' subrange of 6.5-20 MHz.

According to literature [16], the ultrasound extinction cross-section, σ_e, was fitted in a relevant range of ultrasound frequencies (from 6.5 MHz to 20 MHz) by Equation (S1):

$$\sigma_e(R_0;\Omega) = 4\pi R_0^2 \frac{c_0\delta_\eta}{R_0\omega_0} \frac{\Omega^2}{(1-\Omega^2)^2 + {\delta_\eta}^2\Omega^2} \qquad \text{(S1)}$$

Where $\Omega = \frac{\omega}{\omega_0}$ is the ultrasound frequency normalized to the resonance frequency ω_o; c_o is the ultrasound phase velocity in MilliQ water (1476 m/s); R_0 is the mean radius of the MBs; δ is the viscous damping coefficient. The best-fit values of ω_o and δ are reported in **Table S1.** The values are well-consistent with the ones obtained in literature [16] for echogenic PVA-MBs. This indicates that the Y-carrier, cRGD-PVA-DOTA MBs, is echogenic and did not undergo significant perturbation in passing through the neurointerventional catheter.

Table S1. Resonance frequency and viscous damping coefficient values of cRGD-PVA-DOTA MBs in water dispersion that have passed through the neurointerventional catheter, as fitted according to the best-fit model (Equation (S1)).

ω_0 (MHz)	δ_η
13.5 ± 0.2	2.0 ± 0.1

Details of the adhesion experiments on HUVEC cells. Once demonstrated that it is possible to efficiently co-load both y-chelator DOTA and the molecular targeting ligand cRGD onto the same PVA MBs surface, an experiment was performed to establish the binding ability of the carrier, cRGD-PVA-DOTA MBs, on HUVEC cells in a fluid channel. HUVECs were grown into Ibidi μ-Slide I 0.4 microchannel (external dimensions 25.5 mm × 75.5 mm, channel length 50 mm, width 5 mm, height 0.4 mm, cell growth area 2.5 cm^2, total channel volume is 100 μL and that of the 60 μL reservoirs). Specifically, each channel was filled with a 2% bovine gelatin solution and left to dry for 2 hours, after which 10 washing steps in sterile PBS were performed. The gelatin present on the inner walls of the microchannel promotes cell adhesion and growth. For a typical flow experiment approximately 10^5 cells were placed inside the channel which is immediately turned over and left overnight in the incubator to allow the cells to adhere to the upper wall of the channel. The following day, the channel was washed 3 times with culture medium, previously thermostated at 37°C, to eliminate cell residues that had not adhered to the surface of the channel. The main result is shown in Figure 6 of the manuscript and corroborates the hypothesis that cRGD-PVA-DOTA MBs may be used *via* catheter for targeting $\alpha_v\beta_3$ integrins in GBM-associated vasculature endothelium.
